# Genomic Dissection of Leaf Angle in Maize (*Zea mays* L.) Using a Four-Way Cross Mapping Population

**DOI:** 10.1371/journal.pone.0141619

**Published:** 2015-10-28

**Authors:** Junqiang Ding, Luyan Zhang, Jiafa Chen, Xiantang Li, Yongming Li, Hongliang Cheng, Rongrong Huang, Bo Zhou, Zhimin Li, Jiankang Wang, Jianyu Wu

**Affiliations:** 1 College of Agronomy, Synergetic Innovation Center of Henan Grain Crops and National Key Laboratory of Wheat and Maize Crop Science, Henan Agricultural University, Zhengzhou, China; 2 The National Key Facility for Crop Gene Resources and Genetic Improvement, Institute of Crop Science and CIMMYT China Office, Chinese Academy of Agricultural Sciences, Beijing, China; China Agricultural University, CHINA

## Abstract

Increasing grain yield by the selection for optimal plant architecture has been the key focus in modern maize breeding. As a result, leaf angle, an important determinant of plant architecture, has been significantly improved to adapt to the ever-increasing plant density in maize production over the past several decades. To extend our understanding on the genetic mechanisms of leaf angle in maize, we developed the first four-way cross mapping population, consisting of 277 lines derived from four maize inbred lines with varied leaf angles. The four-way cross mapping population together with the four parental lines were evaluated for leaf angle in two environments. In this study, we reported linkage maps built in the population and quantitative trait loci (QTL) on leaf angle detected by inclusive composite interval mapping (ICIM). ICIM applies a two-step strategy to effectively separate the cofactor selection from the interval mapping, which controls the background additive and dominant effects at the same time. A total of 14 leaf angle QTL were identified, four of which were further validated in near-isogenic lines (NILs). Seven of the 14 leaf angle QTL were found to overlap with the published leaf angle QTL or genes, and the remaining QTL were unique to the four-way population. This study represents the first example of QTL mapping using a four-way cross population in maize, and demonstrates that the use of specially designed four-way cross is effective in uncovering the basis of complex and polygenetic trait like leaf angle in maize.

## Introduction

Improving yield by selecting optimal plant architecture is the main objective in maize breeding. In the past several decades, the increasing yield of maize hybrid varieties has been driven by the steadily increase in plant density (i.e., from 30,000 plants per hectare in the 1930s to >80,000 plants per hectare currently) [1]. During the same period, plant morphological traits were significantly improved. In particular, leaf angle was regarded as the major determinant of plant architecture, and has become more significantly upright to adapt the high planting density [1,2]. Erect leaves can effectively maximize photosynthesis by reducing shading and maintaining light capture as canopies became more crowded [3–5], which in turn increase yield production in high density cultivation [6–8].

To detect the genetic basis of natural variations of leaf angle in maize, quantitative trait loci (QTL) mapping studies have been conducted in various populations, and a number of QTL for leaf angle were identified to be distributed throughout the genome. Mickelson et al. [9] firstly detected nine QTL responsible for leaf angle in a B73×Mo17 population with 180 recombination inbred lines (RILs). By using two F_2:3_ populations derived from crosses between Zi330×K36 and H21×Mo17, Yu et al. [10] identified two and seven QTL for leaf angle in the two populations, respectively. In an F_2:3_ population developed from the commercial hybrid Yedan 13 (a cross between parental lines Ye478 and Dan340) in China, Lu et al. [11] located six QTL for leaf angle, explaining a total of 41.0% of phenotypic variation. In a QTL study using F_2:3_ derived-lines from Yu82×Sheng137, Ku et al. [12] identified three significant QTL for leaf angle, which explained 37.4% of phenotypic variation; subsequently Ku et al. [13] developed another F_2:3_ population from cross Yu82×Yu87-1, and identify five QTL explaining 60.3% of phenotypic variation for leaf angle. Recently, Chen et al. [14] located ten QTL for leaf angle in F_2:3_ families derived from the cross CY5×YL106, but only two stable QTL were detected in multiple environments. The natural variations in leaf architecture were also identified in connected RIL populations in maize. Tian et al. [15] used NAM population from 25 crosses between diverse inbred lines and B73 to conduct joint linkage mapping for the leaf architecture, and identified thirty small-effect QTL for leaf angle. Recently, three RIL populations developed by crossing the common parent Huangzaosi with other three elite Chinese maize inbred lines (i.e., Huobai, Weifeng322 and Lv28) were applied to identify natural alleles responsible for leaf architecture variation. Single population QTL mapping and joint linkage analysis across the three populations identified 13 and 17 QTL for leaf angle, respectively [16]. The large numbers of QTL were identified in diverse mapping populations to strengthen the understanding of the heredity of leaf angle in maize.

In recent years, a multi-parent advanced generation integrated cross (MAGIC) strategy has been proposed as an alternative option for QTL mapping. The development of multi-parent populations was initially reported in mice [17], and encouraging results have since been reported for mapping and identification of candidate genes for serum cholesterol and coat color traits [18,19]. Since the successful application of MAGIC in animal, researchers have made great advances in utilizing this strategy in a wide range of plant species. A number of MAGIC populations have been developed to determine the genetic architecture as well as identify causative factors in Arabidopsis thaliana [20–22], tomato [23], wheat[24,25], rice [26] and barley [27]. Compared with mapping study in bi-parental populations, MAGIC offers several unique advantages for QTL analysis: (1) more targeted traits can be analyzed based on the selection of parents used to make the multi-parent crosses [26]; (2) potentially more QTL can be detected due to more allelic diversity across the multiple parents [28]; and (3) improved precision and resolution of QTL because of the increased level of recombination [29].

Taking the complex and polygenic inheritance nature of leaf angle into account, we deliberately developed the first set of multi-parental mapping population generated from four contrasting parental lines for leaf angle in maize. Recently, the genetic characteristics of the four-way cross population have been well described and improved methods for multi-allelic linkage mapping and QTL analysis have been developed [30,31]. The purpose of present study is to detect the genetic architecture underlying leaf angle, and further evaluate and validate the genetic effect of QTL using near-isogenic lines (NILs) or with previously reported QTL/genes.

## Results

### Phenotype analysis

The average performance and the descriptive statistics of leaf angle in four-way families along with the four parental lines in two different environments were shown in Table 1. Parent D276 had more compact leaf architecture with an average leaf angle of 7.4, whereas parent A188 displayed more expanded leaf architecture with an average leaf angle of 45.2, followed by D72 and Jiao51 with average leaf angle of 39.2 and 38.2, respectively. As shown in S1 Fig, the four-way family lines were also characterized by a high variation in leaf angle, which ranged from 18.6 to 52.5, with a mean of 33.2 across the two locations (Table 1). Both genotypic components of variance (*σ* ^2^_*g*_) and genotype × environment interaction (*σ* ^2^_*ge*_) were significant under the significance level at 0.01, and the heritability was 0.87 from the combined ANOVA across the two environments. The relatively high heritability indicated that much of the phenotypic variance was genetically controlled in the four-way cross population.

**Table 1:** Mean and standard deviation of leaf angle in parents and the four-way cross population together with variance components and heritability estimates in Zhengzhou and Jiyuan.

Location	D276 (mean±SD)	D72 (mean±SD)	Jiao51 (mean±SD)	A188 (mean±SD)	Four-way population		*σ* ^2^_*g*_	*σ* ^2^_*ge*_	*H*
					(mean±SD)	Range			
Zhengzhou	8.5±1.0	35.3±2.1	39.0±5.0	35.4±3.4	28.1±5.7	16.0–49.1	29.5**	-	0.91
Jiyuan	6.3±1.4	43.2±1.5	37.4±3.2	54.6±7.0	38.3±6.5	20.9–55.8	38.6**	-	0.90
Combined	7.4±2.1	39.2±4.1	38.2±2.4	45.2±10.6	33.2±5.8	18.6–52.5	29.0**	5.1**	0.87

** Significant at *P* = 0.01

### Genetic linkage map

**Fig 1:**
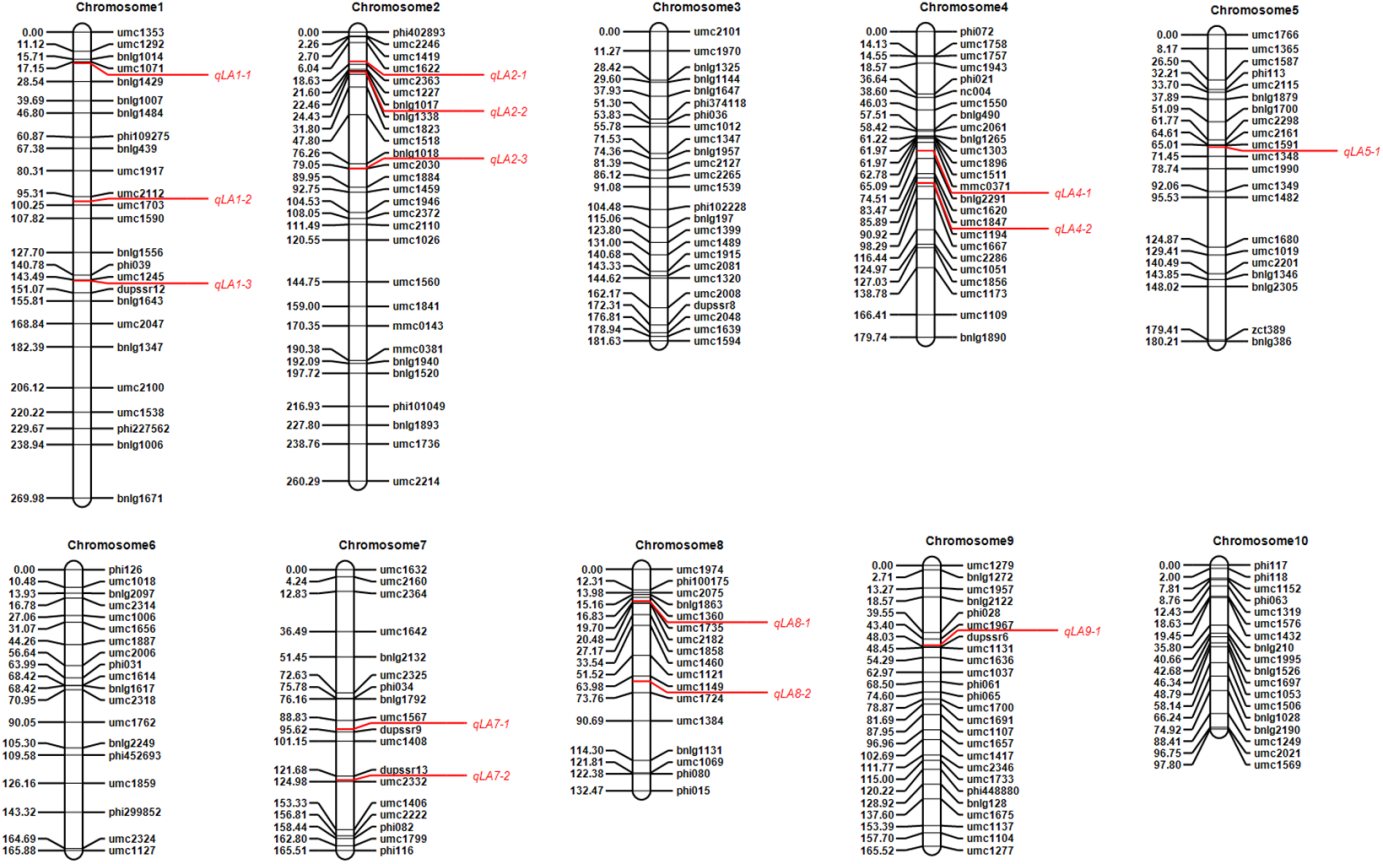
Genetic linkage map and leaf angle QTL identified in four-way cross population.

Among the 222 markers, 83 markers were category ABCD; 62 markers were category A = B; 50 markers were category C = D; 17 markers were category A = CB = D; and 10 markers were category A = DB = C. Marker *umc1319* could not be linked with any marker groups whose category was A = B, and the combined genetic linkage map was constructed by the other 221 markers (Fig 1). There were 25, 28, 25, 25, 21, 19, 18, 17, 25, and 18 markers distributed on the 10 maize chromosomes, respectively (Table 2). The whole length of the genome was 1799.03 cM, with an average marker distance at 8.53 cM.

**Table 2:** Number of markers belonging to the five categories on the 10 chromosomes.

Chrom. No.	Marker category					Total
	ABCD	A = B	C = D	A = CB = D	A = DB = C	
1	8	6	6	2	3	25
2	8	9	9	1	1	28
3	14	5	3	3	0	25
4	8	11	3	3	0	25
5	10	5	4	2	0	21
6	6	6	5	0	2	19
7	7	6	4	1	0	18
8	3	2	8	2	2	17
9	10	8	5	2	0	25
10	9	3	3	1	2	18
Total	83	61	50	17	10	221

The number of markers belonging to each category was shown in Table 2 for the 10 chromosomes. The number of markers belonging to category ABCD was 14 on chromosome 3 which was the largest among all chromosomes, and was 3 on chromosome 8 which was the smallest among all chromosomes. The number of markers belonging to category A = B was 11 on chromosome 4 which was the largest among all chromosomes, and was 2 on chromosome 8 which was the smallest among all chromosomes. The number of markers belonging to category C = D was 9 on chromosome 2 which was the largest among all chromosomes, and was 3 on chromosomes 3, 4 and 10 which was the smallest among all chromosomes. The number of markers belonging to category A = CB = D was 3 on chromosomes 3 and 4 which was the largest among all chromosomes, and was 0 on chromosome 6 which was the smallest among all chromosomes. The number of markers belonging to category A = DB = C was 3 on chromosome 1 which was the largest among all chromosomes, and was 0 on chromosomes 3, 4, 5, 7 and 9 which was the smallest among all chromosomes.

Marker orders on maps of the two single crosses (i.e., AB and CD) were the same as that in the combined map (S2 and S3 Figs). However, the AB map did not contain markers of category A = B, and the CD map did not contain markers of category C = D. Lengths of AB and CD maps were 1810.13 cM and 1630.98 cM, which contained 160 and 171 markers, respectively. The 10 AB chromosomes (S2 Fig) had 19, 19, 20, 14, 16, 13, 12, 15, 17 and 15 markers, respectively, and the 10 CD chromosomes (S3 Fig) had 19, 19, 22, 22, 17, 14, 14, 9, 20 and 15 markers, respectively. Taking chromosome 1 as an example, the length of combined map was 269.98 cM with 25 markers. Six among the 25 markers were category A = B which were not on the AB map. The length of chromosome 1 on the AB map was 278.70 cM with 19 markers. Six among the 25 markers were category C = D which were not on the CD map. The length of chromosome 1 on the CD map was 221.13 cM with 19 markers.

### QTL mapping of leaf angle

The average marker distance on the combined linkage map was 8.53 cM, close to 10 cM. So the number of independent tests (*M*_*eff*_) was about 0.072 times the genome length under the genome-wide type I error rate (*α*_*g*_) at 0.05 [30,32]. LOD threshold was set at 3.97 which was derived from the empirical formula under *α*_*g*_ = 0.05. The information of significant QTL detected from individual environment and across the two environments was summarized in S1 and S2 Tables, and Table 3, which included QTL position, LOD score, genetic effects (additive effects *a*_*F*_ and *a*_*M*_, and dominant effect *d*), phenotypic variation explained (PVE) and the mean value of four different genotypes. Twelve and 11 QTL were detected in Jiyuan and Zhengzhou environments, respectively (S1 and S2 Tables). A total of 14 QTL affecting leaf angle were detected across the two environments, which included three each on chromosomes 1 and 2, two each on chromosomes 4, 7 and 8, and one each on chromosomes 5 and 9 (Table 3). Positions of leaf angle QTL detected across the two environments are marked in linkage maps (Fig 1). Single QTL detected in four-way cross population can explain from 2.27% to 7.75% of the phenotypic variation. The difference of QTL effect was also observed. For the first additive effect (*a*_*F*_) of the 14 QTL, five of them were detected with positive *a*_*F*_, and the other nine were detected with negative *a*_*F*_. For the second additive effect (*a*_*M*_), eight QTL had positive *a*_*M*_, and the other six had negative *a*_*M*_. Seven QTL had positive *d*, and the other seven had negative *d*. Estimated values of genotypic means were also shown in Table 2, which could be used to determine the favorable genotypes and alleles. Taking *qLA1-1* as an example, genotype *AD* had the smallest leaf angle among the four genotypes *AC*, *AD*, *BC* and *BD*. Therefore, the genotypic combination of two alleles from parents A (i.e., D276) and D (i.e., Jiao51) were the favorable genotype at this locus.

**Table 3:** Estimated QTL locations and genetic effects affecting leaf angle using average data from two environments.

QTL	Chrom. bin	Position (cM)	Left marker	Right marker	LOD score	Genetic effects^a^			PVE (%)^b^	Genotypic mean			
						*a*_*F*_	*a*_*M*_	*d*		*A*_*q*_ *C*_*q*_	*A*_*q*_ *D*_*q*_	*B*_*q*_ *C*_*q*_	*B*_*q*_ *D*_*q*_
*qLA1-1*	1.01/02	18	umc1071	bnlg1429	4.67	-0.74	0.43	0.27	2.27	32.49	31.10	33.44	33.10
*qLA1-2*	1.04/05	98	umc2112	umc1703	7.76	-1.06	0.53	0.16	4.10	32.19	30.83	34.01	33.27
*qLA1-3*	1.07/08	144	umc1245	dupssr12	9.77	-1.28	0.18	0.04	4.88	31.45	30.99	33.93	33.65
*qLA2-1*	2.01/02	17	umc1622	umc2363	7.87	-1.12	-0.02	0.28	4.06	31.56	31.06	33.25	33.85
*qLA2-2*	2.02	23	bnlg1017	bnlg1338	13.72	-0.05	-1.54	0.20	7.23	31.12	33.79	30.82	34.30
*qLA2-3*	2.04	79	bnlg1018	umc2030	9.26	-0.29	1.23	0.11	4.54	33.54	30.86	33.90	31.66
*qLA4-1*	4.06	70	mmc0371	bnlg2291	12.54	-1.07	1.40	-0.14	7.75	32.76	30.23	35.17	32.10
*qLA4-2*	4.07	89	umc1847	umc1194	5.14	-1.14	0.28	-0.18	3.77	31.50	31.30	34.14	33.23
*qLA5-1*	5.04	66	umc1591	umc1348	10.64	0.04	1.34	-0.01	5.44	33.88	31.23	33.84	31.12
*qLA7-1*	7.02/03	94	umc1567	dupssr9	10.81	-1.37	0.07	-0.06	5.66	31.16	31.15	34.01	33.77
*qLA7-2*	7.04	124	dupssr13	umc2332	4.79	0.11	-0.89	-0.06	2.38	31.68	33.57	31.58	33.24
*qLA8-1*	8.03	19	umc1360	umc1735	6.04	0.23	-0.98	0.07	3.06	31.84	33.67	31.24	33.35
*qLA8-2*	8.06	67	umc1149	umc1724	12.21	1.46	-0.52	-0.22	6.99	33.26	34.73	30.77	31.38
*qLA9-1*	9.01/02	47	umc1967	dupssr6	6.40	0.17	-1.08	-0.03	3.42	31.58	33.81	31.31	33.40

^a^: The genetic effects of *a*_*F*_ and *a*_*M*_ were the additive genetic effects of the two single crosses, D276×D72 and A188×Jiao51, respectively; the genetic effect of *d* was the dominant effect between the two single crosses.

^b^: Phenotypic variation explained.

### QTL validation using NIL populations

To validate the genetic effects of the favorable alleles from D276, we selected two of 14 QTL detected in four-way population, *qLA2-1* and *qLA4-2* at which *a*_*F*_ was negative, and NILs segregating at the target loci were developed (Table 4). To verify the genetic effect of *qLA2-1*, we developed one pair of NILs, M02-1-1 and M02-1-2, which carried the homozygous alleles of *qLA2-1* from parental line D276 and D72, respectively. The genetic background of M02-1-1 and M02-1-2 was screened with 102 SSR markers randomly distributed on the maize genome and no genetic difference were observed since they both derived from self-cross progenies of the same BC_4_F_1_ plant (data not shown). However, M02-1-1 had more upright orientation with a leaf angle of 28.5 while M02-1-2 displayed more horizontal orientation with a leaf angle of 47.5. The difference of leaf angle in the set of NILs showed that target QTL (*qLA2-*1) can significantly improve the leaf angle as expected. Similar results were achieved in the set of NILs (M04-2-1 and M04-2-2) segregating at *qLA4-2*. The line M04-2-1 showed more compact leaf architecture with the leaf angle of 29.8 while M04-2-2 displayed more extended leaf architecture with the leaf angle of 40.9.

**Table 4:** Genetic effects of QTL for leaf angle identified in NIL populations.

QTL	Favorable alleles^a^	NILs	Target region^b^	Leaf angle	Effect of target QTL
*qLA2-1*	D276	M02-1-1	+/+	28.5	19.0**
		M02-1-2	-/-	47.5	
*qLA4-2*	D276	M04-2-1	+/+	29.8	11.1**
		M04-2-2	-/-	40.9	
*qLA4-1*	Jiao51	M04-1-1	-/-	59.0	11.2**
		M04-1-2	+/+	47.8	
*qLA5-1*	Jiao51	M05-1-1	-/-	57.9	9.3**
		M05-1-2	+/+	48.6	

^a^: the alleles from the given parent can decrease the leaf angle

^b^: +/+ indicate the target QTL region of NIL is homozygous alleles from D276 or Jiao51

-/-: indicate the target region of NIL is homozygous alleles from counterpart parents (D72 or A188)

** significant at *P* = 0.01

To verify the genetic effects of favorable alleles from Jiao51, one pair of NILs (M04-1-1 and M04-1-2) segregating at *qLA4-1* was produced. As shown in Table 4, significant difference of leaf angle (11.2) was observed between M04-1-1 and M04-1-2. Similarly, significant difference of leaf angle (9.3) was also observed in the other set of NILs (M05-1-1 and M05-1-2) segregating at *qLA5-1*. Since there were no detected difference of genetic background between NILs genotyped by 102 SSR markers randomly distributed on the maize genome (data not shown), these results indicated that the target QTL had a significant effect for leaf angle in the NIL background, which was in line with the effects detected in the four-way cross population.

## Discussion

### QTL mapping using four-way crosses population

In plant species, most QTL studies have been conducted in populations initiated from crosses between two inbred lines. As an alternative method to traditional QTL mapping in bi-parental populations, four-way cross design is a more economical strategy since it provides tests for QTL segregation in four lines simultaneously in one experiment. Moreover, the strategy can potentially increase the probability of detecting QTL if they segregate in one line cross but not in the other [33]. Considering the complex and polygenetic nature of leaf angle in maize, four-way population can be expected to better dissect the genetic basis of leaf angle in maize. Compared with the results of only several QTL of leaf angle identified in a single bi-parental population [13], the number of QTL and alleles within them is greatly increased in current studies, which indicates the utilization of four-way cross population is effective in uncovering the genetic basis of polygenetic agronomic trait like leaf angle in maize.

In contrast to a simple line cross in which only two alleles are involved, a four-way cross can have a maximum of four alleles. Because of this, the additive and dominant effects in a four-way cross are defined differently from a simple cross to accommodate different inbred cross designs [33]. In this study, we inherited the definition of genetic effects in [31], i.e., we defined two additive effects *a*_*F*_, *a*_*M*_ and one dominant effect *d*. Denote *μ*_1_,⋯,*μ*_4_ as mean performances (or genotypic values) of the four QTL genotypes *A*_*q*_ *C*_*q*_, *A*_*q*_ *D*_*q*_, *B*_*q*_ *C*_*q*_ and *B*_*q*_ *D*_*q*_ at locus *q*, then the genetic effects were defined as following: $a_{F}=\frac{1}{4}(\mu_{1}+\mu_{2}-\mu_{3}-\mu_{4})$, $a_{M}=\frac{1}{4}(\mu_{1}-\mu_{2}+\mu_{3}-\mu_{4})$, and $d=\frac{1}{4}(\mu_{1}-\mu_{2}-\mu_{3}+\mu_{4})$ [30]. *a*_*F*_ is the additive effect in the first single cross F_1_ (i.e., AB), which reflects the difference between the performance of alleles *A* and *B* based on the alleles *C* and *D* in this four-way cross design. In other words, if *a*_*F*_ is positive, it means that allele *A* can achieve larger phenotypic value than allele *B* in the design (A×B)×(C×D). Similarly, *a*_*M*_ is the additive effect in the second single cross F_1_ (i.e., CD), which reflects the difference between the performance of alleles *C* and *D* based on the alleles *A* and *B* in this four-way cross design. In other words, if *a*_*M*_ is positive, it means that allele *C* can achieve larger phenotypic value than allele *D* in the design (A×B)×(C×D). And *d* reflects the dominant effect between the two single crosses.

In this study, phenotypic value of the four-way F_1_ was represented by the average trait values of their selfing progeny family. Additive effect calculated by this way was the same as the one calculated by phenotypic value of four-way F_1_ directly. However, dominant effect was only half of that calculated by the F_1_. For example if the genotype of four-way F_1_ was *AC*, we assumed the additive effect was *a*, the dominant effect was *d*, and the mid-parent value was *μ*. Genotypes of F_2_ were *AA*, *AC* and *CC* with the ratio of 1:2:1. Their genotypic values were *μ*+*a*, *μ*+*d*, and *μ*-*a*, respectively. So the average value was 0.25×(*μ*+*a*)+0.5×(*μ*+*d*)+0.25×(*μ*-*a*) = *μ*+0.5×*d*. The dominant effect was halved.

ICIM was chosen in this study to perform QTL analysis. As indicated in Zhang et al. [30], an inclusive linear model that includes marker variables and marker interactions so as to completely control all genetics effects was proposed and used for genetic background control in ICIM. By extensive simulations and comparisons with simple interval mapping, multiple-QTL models and composite interval mapping, ICIM was illustrated to have higher detection powers, lower false discovery rate, more precise estimates of QTL positions and effects. In this study, QTL analysis was performed for each environment separately, as well as for the mean across environments. A proper multi-environment QTL analysis allows for differential expression of QTL across environments, and allows the correlation between environments to refine the position of QTL. We plan to develop the multi- environment QTL analysis methods for four-way crosses based on ICIM in the future.

### Comparison of the empirical formula with permutation tests

The empirical formula was used to determine LOD threshold in this study. For comparison with the empirical LOD threshold values, permutation tests were also conducted on this population for 10000 times. LOD threshold 4.49 was achieved under *α*_*g*_ = 0.05, which was a little higher than 3.97 from the empirical formula. If threshold 4.49 was taken, all the 14 QTL detected across environments in Table 3 could also be identified. Only one QTL on chromosome 1 detected in Jiyuan environment (S1 Table) and one QTL on chromosome 2 detected in Zhengzhou environment (S2 Table) could not be identified. The reason for the high threshold values may be explained as follows. In permutation tests, the relationship between marker type and phenotype is randomly shuffled so as to generate the scenario of non-QTL situation, i.e., the null hypothesis where there is no genetic variation. It is understood that the phenotype should follow a normal distribution under the null hypothesis. However, the shuffling of the original phenotype cannot change the distribution. If the original phenotype did not follow the normal distribution, neither will the shuffled phenotype. Therefore, the permutation tests in QTL mapping may not generate the non-QTL scenario for non-normally distributed traits. In this study, *P* values of Shapiro-Wilk test for phenotypic values under the three environments (two environments and their means) were 0.31, 0.0001, and 0.09. Phenotypes in the second and third environments didn’t follow the normal distribution under the significance level at 0.1. Here the normal distribution tests were conducted by the univariate (Proc univariate) procedure of SAS software [34].

### Comparison with known QTL and genes from the literature

So far, a number of QTL/genes for leaf angle have been identified in various populations in maize. We compared the published QTL/genes of leaf angle with those detected in present study and consensus QTL/genes were identified. The most prominent region is chromosome 2 (bin 2.01) where *qLA2-1* was detected in the four-way cross population. Within *qLA2-1* region, Moreno et al. [35] cloned a recessive gene *liguleless1* (*lg1*) by mutagenesis in maize, and mutant carrying *lg1* had no ligule or auricle, leading to considerably more upright leaves than their normal counterparts. This region was also repeatedly reported in two separate studies, which consistently identified the common leaf angle QTL overlapping with the location of *lg1* [15,16]. These QTL detected in different mapping populations shared a high congruence, which strongly supported the candidacy of *lg1* for *qLA2-1*. The other consistent QTL across various population included *qLA1-1*, *qLA1-2*, *qLA2-3*, *qLA4-1*, *qLA7-1* and *qLA8-2* (S3 Table). Within *qLA8-2* region, Tian et al. [15] and Li et al. [16] also detected the important QTL by using joint linkage analysis in NAM population and three connected maize populations, respectively. Other common QTL included *qLA1-1* overlapping with the ones detected by Li et al. [16], and *qLA1-2*, *qLA2-3*, *qLA4-1* and *qLA7-1* showing congruency with ones detected by Tian et al. [15]. These common QTL across different mapping populations imply some conservation in the genes responsible for leaf angle, which will be important target regions for molecular-assisted selection (MAS) to improve the plant architecture in maize breeding program.

For the purpose of screening target leaf angle QTL for MAS in maize breeding program, four QTL (i.e., *qLA2-1*, *qLA4-1*, *qLA4-2* and *qLA5-1*) were selected and the genetic effects were validated. Since each QTL has two additive effects (i.e., *a*_*M*_ and *a*_*F*_), we only selected larger additive effect of target QTL and verified them by using NILs. In the NIL background, the effects of the target QTL were precisely evaluated, which showed that each of the four selected QTL had larger additive effect in NILs compared with their counterparts identified in four-way cross population. Moreover, the four QTL showed various LOD value, which varied from 5.14 (close to the LOD threshold 4.49) to 12.54 identified in the four-way cross population (Table 3). These significant effects of target QTL validated in NILs further imply the large amount of leaf angle QTL identified in four-way population are “real” ones despite more QTL remained for further cross-validation.

This study has reported the first four-way cross population in maize and highlighted the potential in application of mapping the complex and polygenetic trait like leaf angle in maize. However, the full power of the current multi-parental population has not been realized due to the relative low marker density. Therefore, compared with the SSR markers used in present study, high-throughput SNP markers are necessary to applied to further improve the precision and power of QTL mapping with increased marker density in the future study.

## Materials and Methods

The experiment was conducted in Zhengzhou Experiment Station (34°51'N 113°35'E) and Jiyuan Experiment Station (35°4'N 112°36'E) of Henan Agricultual University (HAU). At the two experimental locations, HAU has set up experimental field bases for non-profit agricultural research with a wide array of partners in China. In present study, the field experiments in the two experimental stations were approved by HAU. Further, the experimental stations where field studies were conducted are not protected locations for endangered or protected species.

### Plant materials

**Fig 2:**
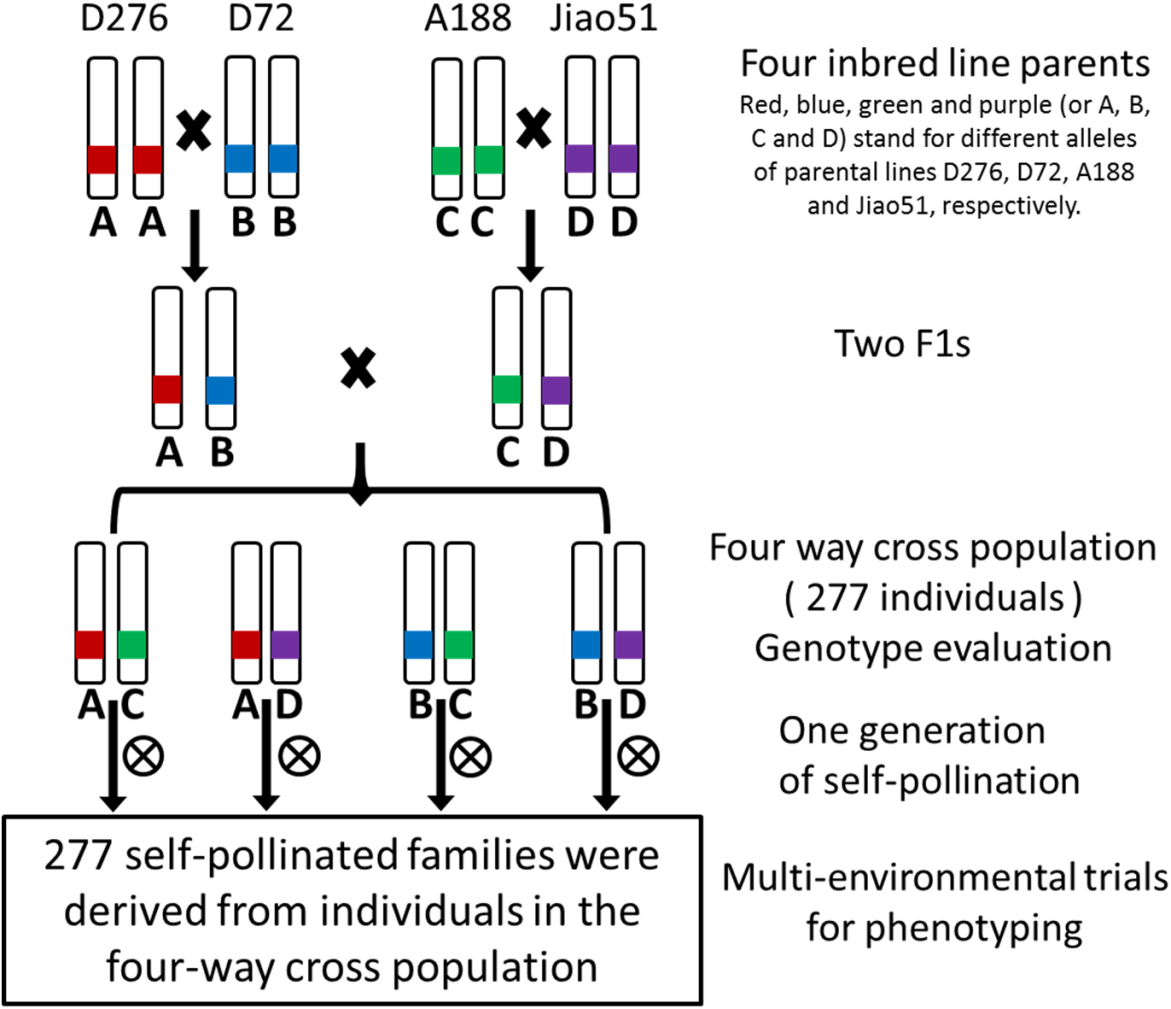
The procedure of developing the four-way cross population.

The procedure of developing four-way cross population was shown in Fig 2. Four maize inbred lines, i.e., D276, D72, A188 and Jiao51 (denoted as A, B, C and D, respectively), were selected as founders of the four-way population based on the agronomic performance for a range of traits in maize breeding program. Two single crosses were firstly made: D276×D72 (AB) and A188×Jiao51 (CD). The two F_1_ hybrids were then intercrossed (AB×CD) to generate 305 ‘four-way’ (ABCD) F_1_, which was self-pollinated to generate enough seeds for field phenotypic evaluation. Due to the poor self-pollination of some ABCD F_1_ lines, enough seeds (>200 kernels) were available from the ears of 277 ABCD F_1_ plants. Therefore, the 277 ABCD F_1_ plants were used for genotyping, and their selfing progenies (here we refer to 277 four-way cross family lines) were used for multi-environmental phenotyping. Four parental lines and two single crosses F_1_ were used to screen polymorphism of SSR (Simple Sequence Repeat) markers, and then the four-way cross F_1_ population (ABCD) plants were genotyped by 222 polymorphic SSR markers.

### Field trials and trait evaluation

In 2011, the 277 four-way family lines, together with their four parents were grown at two locations in central part of China, namely Zhengzhou Experiment Station (34°51'N 113°35'E) and Jiyuan Experiment Station (35°4'N 112°36'E) of Henan Agricultual University (HAU). A randomized complete block design with three replications was applied in each location. Each plot had one row that was 4 m long and 0.67 m wide, with a total of 15 plants at a density of 52,500 plants/ha. The field management followed common agricultural practice in maize production in China. Eight plants in the middle of each plot were chosen to measure the leaf angle 10 days after anthesis. Leaf angle was determined for four leaves above the primary ear as the angle of each leaf from a plane defined by the stalk below the node subtending the leaf [9]. The trait value of each family was acquired from the average of the eight measured plants in each replication.

A set of NILs was developed to validate the genetic effect of target QTL identified in four-way cross population. To verify the first additive effect (*a*_*F*_), NILs were produced by crossing a recurrent parent, D72, with a donor parental line, D276, through four cycles of advanced backcrosses. From the BC_2_F_1_ generation, the QTL region was detected by flanking markers of target loci. In BC_4_F_1_ generation, the individuals with heterozygous target segment were selected to self-cross into homozygous lines, followed by genotyping using an additional 102 SSR markers to estimate the introgressions of non-targeted segments from the donor. Similar procedure was used to develop NILs to verify the second additive effect (*a*_*M*_), in which the A188 and Jiao51 were donor parent and recurrent parent, respectively. In the summer seasons of 2012 and 2013, NILs together with the recurrent parental lines were planted in the Zhengzhou Experiment Station with three replications for phenotyping.

### Phenotypic data analysis

Analysis of variance (ANOVA) for phenotypic data was performed using the General Line Model (Proc GLM) procedure of SAS software [34] which include environment (*e*), genotype (*g*), genotype by environment interaction (*g*×*e*), and replication effects (*r*) in the model, from which the components of variance were estimated. The broad-sense heritability (*H*) across the environments was computed using *H* = *σ* ^2^_*g*_/(*σ* ^2^_*g*_+*σ* ^2^_*ge*_ */e*+*σ* ^2^_*e*_ */er*) according to Knapp et al. [36], where *σ* ^2^_*g*_, *σ* ^2^_*ge*_ and *σ* ^2^_*e*_ represent estimated variances for genetic effects, genotype by environment interaction and the random error, respectively.

### Genetic map construction and QTL mapping

The algorithms for recombination frequency estimation and linkage map construction were proposed by Zhang et al. [31], which was implemented in linkage map construction and QTL mapping software package for clonal F_1_ and four-way crosses (called GACD, Genetic Analysis of Clonal F_1_ and Double cross). Markers were first classified into five categories, i.e., ABCD, A = B, C = D, A = CB = D and A = DB = C. Category ABCD represents the case of fully informative markers where each marker shows four identifiable alleles in the four inbred parents. Other categories provide incomplete information caused by the confounding of genotypes. Category A = B and category C = D represent the cases of male-polymorphic and female-polymorphic markers respectively. Category A = CB = D represents the case of no-polymorphism between parents A and C, and between B and D, but is polymorphic between AC and BD. Category A = DB = C represents the case of no-polymorphism between parents A and D, and between B and C, but is polymorphic between AD and BC [31]. Then the maximum likelihood estimates of recombination frequencies between each two markers were achieved based on the theoretical frequencies of identifiable genotypes under different scenarios. Markers were anchored on the chromosomes by referred to the physical map. A combined algorithm of nearest neighbor and Two-opt algorithm of Traveling Salesman Problem [37] was used to determine the marker order [30].

The algorithm of inclusive composite interval mapping (ICIM) for four-way crosses was used for QTL mapping [30] based on the linkage map we built, which was also implemented in software package GACD. Phenotypic data in both environments as well as the means across environments were analyzed. Stepwise regression was used to select significant marker variables and the two probabilities for entering and removing variables were set at 0.001 and 0.002. The scanning step was set at 1 cM. The empirical formula derived from [30] was used to set the LOD threshold, i.e., LOD threshold should be determined by the formula $LOD=\chi_{\alpha_{p}}^{2}(df)/2ln(10)$. Here $\alpha_{p}=\frac{\alpha_{g}}{M_{eff}}$ is the type-I error per scanning test; *α*_*g*_ is the genome-wide type I error rate; *M*_*eff*_ is the number of independent tests depending on the genome length, marker density and population type; *df* is 3 in QTL mapping of four-way cross populations; and $\chi_{\alpha_{p}}^{2}(df)$ is the inverse *χ* ^2^ distribution that returns the critical value of a right-tailed probability *α*_*p*_ for the degree of freedom *df* [30].

## Supporting Information

S1 Fig The distribution of the leaf angle in single environment as well as across the environments. (TIF)

S2 Fig Linkage maps of the first single cross (AB). (TIF)

S3 Fig Linkage maps of the second single cross (CD). (TIF)

S1 Table Estimated QTL locations and genetic effects affecting leaf angle in Jiyuan, Henan, China. (DOCX)

S2 Table Estimated QTL locations and genetic effects affecting leaf angle in Zhengzhou, Henan, China. (DOCX)

S3 Table Comparison of the present QTL results with previous studies. (DOCX)
