# Supplementary figures and images for: Genomic Dissection of Leaf Angle in Maize (Zea mays L.) Using a Four-Way Cross Mapping Population
Source: PLoS One. 2015 Oct 28;10(10):e0141619. doi: 10.1371/journal.pone.0141619 (PMC4625009; doi:10.1371/journal.pone.0141619)

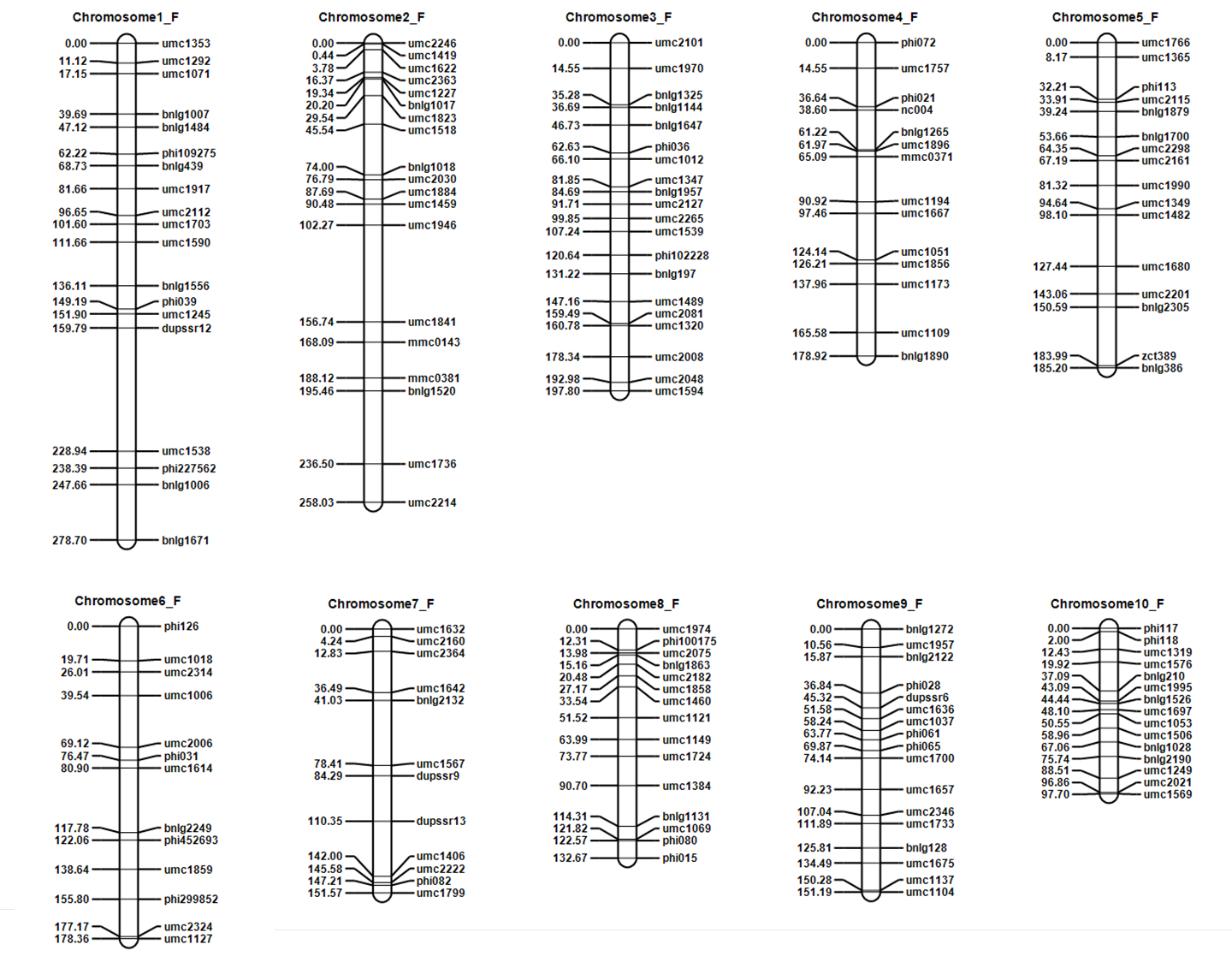

Supplement: S1 Fig — (TIF) [file pone.0141619.s001.tif]

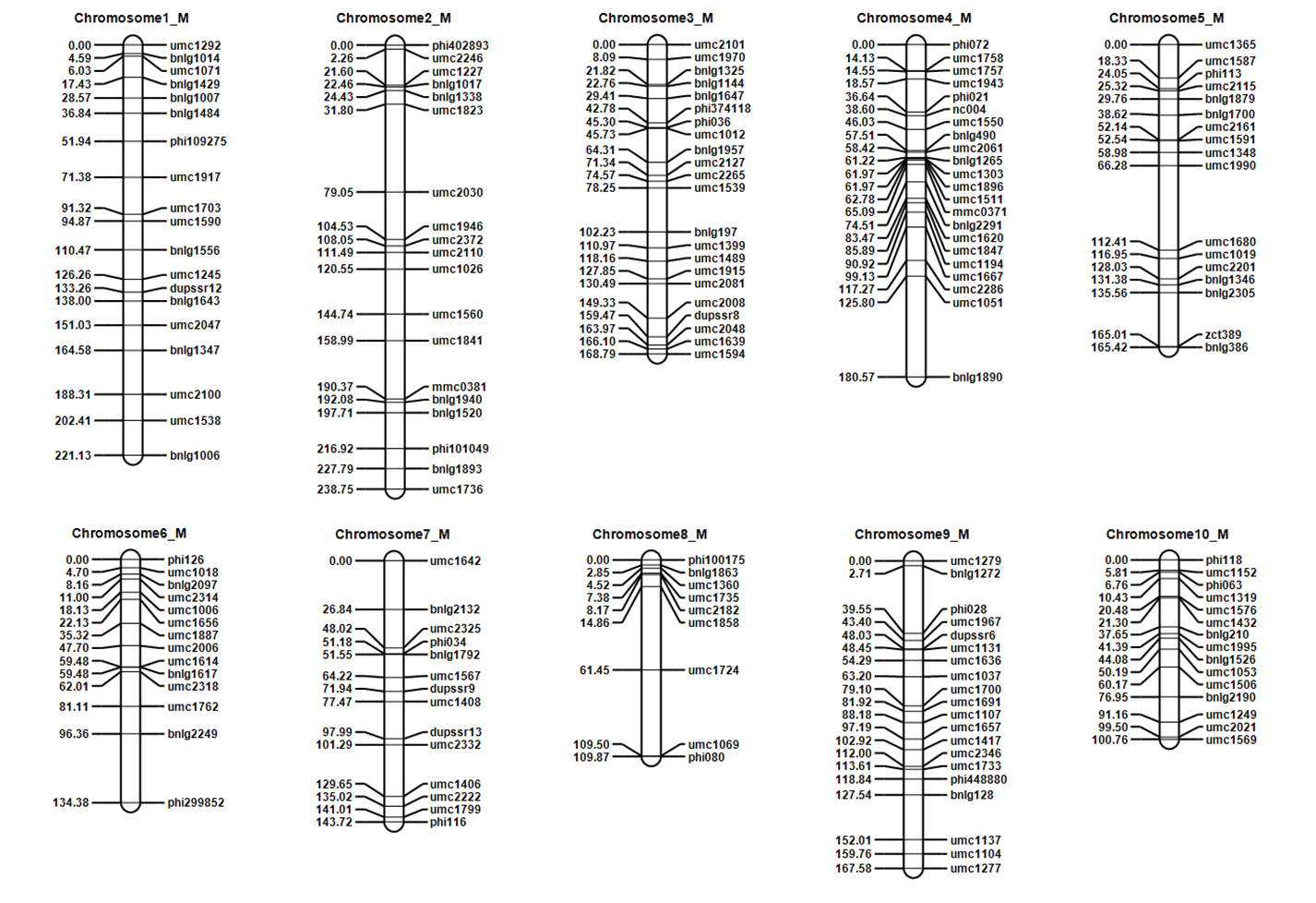

Supplement: S2 Fig — (TIF) [file pone.0141619.s002.tif]

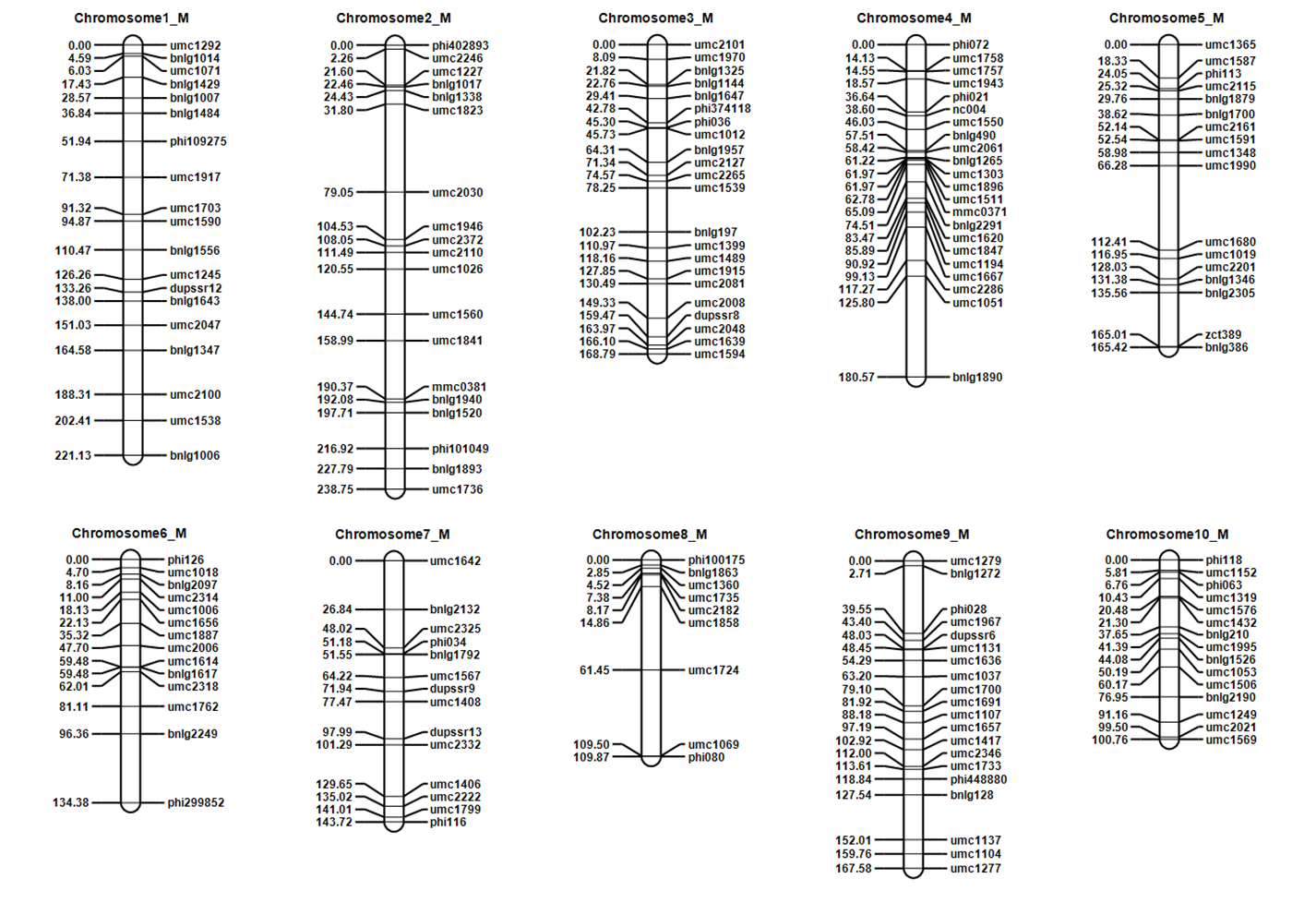

Supplement: S3 Fig — (TIF) [file pone.0141619.s003.tif]
