# Supplementary material for: Genomic Dissection of Leaf Angle in Maize (Zea mays L.) Using a Four-Way Cross Mapping Population
Source: PLoS One. 2015 Oct 28;10(10):e0141619. doi: 10.1371/journal.pone.0141619 (PMC4625009; doi:10.1371/journal.pone.0141619)
Supplement: S1 Table — (DOCX) [file pone.0141619.s004.docx]

**S1 Table. Estimated QTL locations and genetic effects for leaf angle in Jiyuan environment.**

| Chrom. bin | Position (cM) | Left marker | Right marker | LOD score | Genetic effects^a^ | | | PVE (%)* | Genotypic mean | | | |
| --- | --- | --- | --- | --- | --- | --- | --- | --- | --- | --- | --- | --- |
|  |  |  |  |  | *a*_F_ | *a*_M_ | *d* |  | *A_q_C_q_* | *A_q_D_q_* | *B_q_C_q_* | *B_q_D_q_* |
| 1.03 | 59 | bnlg1484 | phi109275 | 5.46 | -1.10 | 0.56 | -0.02 | 3.49 | 37.44 | 36.36 | 39.69 | 38.52 |
| 1.04 | 95 | umc1917 | umc2112 | 4.15 | -0.95 | 0.21 | 0.21 | 2.11 | 37.43 | 36.60 | 38.92 | 38.92 |
| 1.07/08 | 144 | umc1245 | dupssr12 | 7.51 | -1.30 | 0.06 | -0.06 | 3.96 | 36.71 | 36.72 | 39.44 | 39.21 |
| 2.01 | 19 | umc2363 | umc1227 | 7.87 | -1.27 | -0.17 | -0.06 | 4.11 | 36.51 | 36.97 | 39.17 | 39.40 |
| 2.02 | 33 | umc1823 | umc1518 | 13.38 | -0.04 | -1.89 | -0.05 | 8.39 | 36.02 | 39.90 | 36.19 | 39.88 |
| 2.04 | 79 | bnlg1018 | umc2030 | 11.16 | -0.16 | 1.62 | 0.07 | 6.01 | 39.52 | 36.15 | 39.71 | 36.61 |
| 4.05/06 | 65 | umc1511 | mmc0371 | 14.15 | -1.55 | 1.19 | 0.23 | 8.52 | 37.89 | 35.05 | 40.54 | 38.60 |
| 4.08/09 | 124 | umc2286 | umc1051 | 7.66 | -1.33 | 0.28 | 0.46 | 4.71 | 37.42 | 35.94 | 39.15 | 39.52 |
| 5.03/04 | 64 | umc2298 | umc2161 | 6.26 | 0.03 | 1.21 | -0.06 | 3.43 | 39.20 | 36.92 | 39.27 | 36.73 |
| 7.03 | 96 | dupssr9 | umc1408 | 9.84 | -1.49 | -0.08 | -0.14 | 5.41 | 36.32 | 36.76 | 39.58 | 39.47 |
| 7.04/05 | 125 | umc2332 | umc1406 | 4.49 | -0.22 | -0.94 | -0.18 | 2.26 | 36.66 | 38.91 | 37.48 | 38.99 |
| 8.06 | 68 | umc1149 | umc1724 | 14.08 | 1.84 | -0.69 | -0.19 | 8.88 | 38.98 | 40.75 | 35.68 | 36.68 |

^a^: the genetic effects of *a_F_* and *a_M_* were the additive genetic effects of the two single crosses, D276×D72 and A188×Jiao51, respectively; the genetic effect of *d* was the dominance effect between the two single crosses.

* Phenotypic variation explained.
