# Supplementary material for: Genomic Dissection of Leaf Angle in Maize (Zea mays L.) Using a Four-Way Cross Mapping Population
Source: PLoS One. 2015 Oct 28;10(10):e0141619. doi: 10.1371/journal.pone.0141619 (PMC4625009; doi:10.1371/journal.pone.0141619)
Supplement: S2 Table — (DOCX) [file pone.0141619.s005.docx]

**S2 Table. Estimated QTL locations and genetic effects for leaf angle in Zhengzhou Environment.**

| Chrom bin | Position (cM) | Left marker | Right marker | LOD score | Genetic effects^a^ | | | PVE (%)* | Genotypic mean | | | |
| --- | --- | --- | --- | --- | --- | --- | --- | --- | --- | --- | --- | --- |
|  |  |  |  |  | *a*_F_ | *a*_M_ | *d* |  | *A_q_C_q_* | *A_q_D_q_* | *B_q_C_q_* | *B_q_D_q_* |
| 1.03 | 61 | phi109275 | bnlg439 | 9.80 | -0.54 | 1.37 | 0.32 | 6.84 | 30.20 | 26.81 | 30.63 | 28.53 |
| 1.07/08 | 143 | phi039 | umc1245 | 7.70 | -1.35 | 0.09 | 0.12 | 5.49 | 27.97 | 27.53 | 30.41 | 30.47 |
| 1.11/12 | 220 | umc2100 | umc1538 | 6.55 | -0.49 | -1.05 | -0.24 | 4.43 | 27.36 | 29.95 | 28.82 | 30.44 |
| 2.01 | 18 | umc1622 | umc2363 | 11.95 | -1.64 | -0.06 | 0.20 | 8.66 | 27.55 | 27.28 | 30.43 | 30.95 |
| 2.02 | 23 | bnlg1017 | bnlg1338 | 11.54 | 0.07 | -1.65 | 0.20 | 8.49 | 27.72 | 30.62 | 27.19 | 30.88 |
| 4.08 | 106 | umc1667 | umc2286 | 6.00 | -0.96 | 1.28 | 0.03 | 6.00 | 29.42 | 26.81 | 31.27 | 28.78 |
| 5.04/05 | 74 | umc1348 | umc1990 | 15.00 | 1.14 | 1.48 | 0.42 | 13.90 | 32.05 | 28.24 | 28.92 | 26.80 |
| 7.01 | 6 | umc2160 | umc2364 | 5.47 | -1.28 | 0.67 | 0.09 | 4.15 | 28.54 | 27.01 | 30.92 | 29.77 |
| 8.03 | 19 | umc1360 | umc1735 | 11.02 | 0.77 | -1.46 | 0.06 | 8.20 | 28.49 | 31.28 | 26.82 | 29.86 |
| 9.03/04 | 82 | umc1691 | umc1107 | 5.73 | 0.28 | -1.14 | -0.28 | 3.86 | 28.07 | 30.91 | 28.06 | 29.80 |
| 9.07 | 136 | bnlg128 | umc1675 | 4.19 | -0.77 | -0.38 | 0.39 | 3.05 | 28.24 | 28.22 | 29.01 | 30.54 |

^a^: the genetic effects of *a_F_* and *a_M_* were the additive genetic effects of the two single crosses, D276×D72 and A188×Jiao51, respectively; the genetic effect of *d* was the dominance effect between the two single crosses.

* Phenotypic variation explained.
