# Supplementary material for: Genomic Dissection of Leaf Angle in Maize (Zea mays L.) Using a Four-Way Cross Mapping Population
Source: PLoS One. 2015 Oct 28;10(10):e0141619. doi: 10.1371/journal.pone.0141619 (PMC4625009; doi:10.1371/journal.pone.0141619)
Supplement: S3 Table — (DOCX) [file pone.0141619.s006.docx]

**S3 Table. Compare the QTL identified in present study with reported genes/QTL from the literature.**

| QTL | Chromosome bin | Left marker | Right marker | Reported genes/QTL |
| --- | --- | --- | --- | --- |
| *qLA1-1* | 1.01/02 | umc1071 | bnlg1429 | *qLA1a*[16] |
| *qLA1-2* | 1.04/05 | umc2112 | umc1703 | m82[15] |
| *qLA1-3* | 1.07/08 | umc1245 | dupssr12 |  |
| *qLA2-1* | 2.01/02 | umc1622 | umc2363 | *lg1*[37], *qLA2a*[16], m189[15] |
| *qLA2-2* | 2.02 | bnlg1017 | bnlg1338 |  |
| *qLA2-3* | 2.04 | bnlg1018 | umc2030 | m200[15] |
| *qLA4-1* | 4.06 | mmc0371 | bnlg2291 | m499[15] |
| *qLA4-2* | 4.07 | umc1847 | umc1194 |  |
| *qLA5-1* | 5.04 | umc1591 | umc1348 |  |
| *qLA7-1* | 7.02/03 | umc1567 | dupssr9 | m804[15] |
| *qLA7-2* | 7.04 | dupssr13 | umc2332 |  |
| *qLA8-1* | 8.03 | umc1360 | umc1735 |  |
| *qLA8-2* | 8.06 | umc1149 | umc1724 | *qLA8*[16], m920[15] |
| *qLA9-1* | 9.01/02 | umc1967 | dupssr6 |  |
